# Expression of Renin-Angiotensin System Components in the Taste Organ of Mice

**DOI:** 10.3390/nu11092251

**Published:** 2019-09-19

**Authors:** Noriatsu Shigemura, Shingo Takai, Fumie Hirose, Ryusuke Yoshida, Keisuke Sanematsu, Yuzo Ninomiya

**Affiliations:** 1 Section of Oral Neuroscience, Graduate School of Dental Sciences, Kyushu University, 3–1–1 Maidashi, Higashi-ku, Fukuoka 812–8582, Japan; takai.shingo.774@m.kyushu-u.ac.jp (S.T.); f-hirose@dent.kyushu-u.ac.jp (F.H.); yoshida.ryusuke@okayama-u.ac.jp (R.Y.); sanematu@dent.kyushu-u.ac.jp (K.S.); 2 Division of Sensory Physiology, Development Center for Five-Sense Devices, Kyushu University, Fukuoka 819-0395, Japan; 3 Section of Orthodontics and Dentofacial Orthopedics, Division of Oral Health, Growth, and Development, Faculty of Dental Science, Kyushu University, Fukuoka 812–8582, Japan; 4 Department of Oral Physiology, Graduate School of Medicine, Dentistry and Pharmaceutical Sciences, Okayama University, Okayama 700-8525, Japan; 5 Monell Chemical Senses Center, Philadelphia, PA 19104, USA

**Keywords:** taste, sodium taste, renin, angiotensin II, angiotensinogen, angiotensin-converting enzyme

## Abstract

The systemic renin-angiotensin system (RAS) is an important regulator of body fluid and sodium homeostasis. Angiotensin II (AngII) is a key active product of the RAS. We previously revealed that circulating AngII suppresses amiloride-sensitive salt taste responses and enhances the responses to sweet compounds via the AngII type 1 receptor (AT1) expressed in taste cells. However, the molecular mechanisms underlying the modulation of taste function by AngII remain uncharacterized. Here we examined the expression of three RAS components, namely renin, angiotensinogen, and angiotensin-converting enzyme-1 (ACE1), in mouse taste tissues. We found that all three RAS components were present in the taste buds of fungiform and circumvallate papillae and co-expressed with αENaC (epithelial sodium channel α-subunit, a salt taste receptor) or T1R3 (taste receptor type 1 member 3, a sweet taste receptor component). Water-deprived mice exhibited significantly increased levels of renin expression in taste cells (*p* < 0.05). These results indicate the existence of a local RAS in the taste organ and suggest that taste function may be regulated by both locally-produced and circulating AngII. Such integrated modulation of peripheral taste sensitivity by AngII may play an important role in sodium/calorie homeostasis.

## 1. Introduction

The renin-angiotensin system (RAS) is a major hormone system involved in body fluid and sodium homeostasis [1]. Angiotensin II (AngII), an octapeptide hormone, is the most powerful biologically active product of the RAS and plays important roles in the regulation of vascular tone, cardiac function, and renal sodium re-absorption. AngII is also thought to be a potent stimulator of sodium appetite and preference. For example, intracerebroventricular or intravenous infusion of AngII in the rat produces dose-dependent salt appetite and stimulates sodium intake over a range of concentrations that are normally rejected [2,3]. The gustatory system provides critical information about the quality and nutritional value of food before it is ingested. Thus, changes in sodium taste sensitivity might contribute to the ingestive behaviors induced by AngII. We recently addressed this hypothesis and revealed that AngII suppresses amiloride-sensitive salt taste responses and enhances the responses to sweet compounds via the AngII type 1 receptor (AT1) expressed in taste cells, without any effects on the amiloride-insensitive salt, sour, bitter, or umami responses [4]. These results suggest that the taste organ is a peripheral target of AngII and that AngII may function to increase sodium intake through the specific reduction in amiloride-sensitive salt taste sensitivity and increase energy intake through the enhancement of sweet responses. However, the molecular mechanisms underlying the modulation of taste function by AngII remain uncharacterized.

It is generally known that AngII is produced by the classical or circulating RAS [1]. Renin is a proteolytic enzyme released primarily from the juxtaglomerular cells of the kidney in response to a decrease in arterial blood pressure or sodium chloride level in the nephron [5]. Angiotensinogen is secreted constitutively, mainly by hepatic cells, into the circulation. Renin cleaves angiotensinogen at the N-terminus to form the decapeptide, angiotensin I (AngI). AngI is converted to AngII through the removal of two C-terminal residues by circulating angiotensin-converting enzyme-1 (ACE1), which is found in various organs, including the lung and kidney [6]. AT1, the main receptor subtype for circulating AngII, is widely distributed throughout the body including vascular smooth muscle, kidney, heart, brain, and taste organ [4,7]. AngII is degraded into smaller active peptides, AngIII, AngIV, and Ang (1–7), by endopeptidases or carboxypeptidases such as ACE2, which is a homolog of ACE1 [8].

In addition to the circulating RAS, it is now recognized that tissues such as the kidneys, brain, heart, adrenal glands and vasculature each have an organ-specific RAS [9,10]. For example, all the RAS components are present in the kidneys, and intrarenal AngII is produced independently of the circulating RAS to function as a paracrine factor via AT1. Inappropriate activation of the intrarenal RAS contributes to the pathogenesis of hypertension and renal injury [11,12]. RAS components are also present in cardiac myocytes and fibroblasts, where they synthesize AngII intracellularly [13,14]. Hyperglycemia selectively upregulates the intracellular RAS system in cardiac myocytes and vascular smooth muscle cells [15], and this is associated with cardiomyocyte apoptosis, oxidative stress, and fibrosis in diabetic rats [16]. The above findings raise the possibility that an organ-specific RAS might also exist in taste tissues and that AngII might be produced locally in response to changes in the peripheral oral environment (e.g., a change in the sodium concentration or osmolality of the saliva or the presence of chemical compounds in foods).

To explore this possibility, we utilized reverse transcription-polymerase chain reaction (RT-PCR), in situ hybridization, and double-staining immunohistochemistry to investigate the expression patterns of renin, angiotensinogen, and ACE in the taste tissues of mice under hydrated and dehydrated conditions.

## 2. Materials and Methods

All experimental procedures were performed in accordance with the Guidelines for the Care and Use of Laboratory Animals established by the National Institutes of Health and approved by the Committee for Laboratory Animal Care and Use at Kyushu University, Japan. The ethical approval code for the animal experiments was A27-009.

### 2.1. Animals

This study used male and female C57BL/6NCrj mice (B6; Charles River, Tokyo, Japan), taste receptor type 1 member 3 (T1R3)-green fluorescent protein (GFP) mice [17] and glutamate decarboxylase 67 (GAD67)-GFP mice [18] aged 8–16 weeks of age and weighing 21–30 g. The mice were housed at a constant temperature (24 ± 1 °C) under a 12 h–12 h light-dark cycle (lights on at 08:00) and given access to food and water ad libitum.

### 2.2. Reverse Transcription-Polymerase Chain Reaction (RT-PCR)

RT-PCR was performed as described previously [4,19,20,21]. Mouse taste buds in the peeled epithelium were individually removed from fungiform or circumvallate papillae by aspiration with a transfer pipette. The RNeasy Plus Micro kit (Qiagen, Stanford, CA, USA) was used to purify RNAs from 100 fungiform or circumvallate taste buds from three mice or from a 1 mm × 1 mm block of epithelial tissue without taste buds. cDNAs were synthesized by RT [oligo(dT)12–18 primer] using the SuperScript pre-amplification system (Invitrogen, Carlsbad, CA, USA). Two protocols were used to prevent genomic DNA from contributing to the signal: (1) primers were chosen to span one or more introns to distinguish the PCR products from genomic DNA; The primer sequences are shown in Supplementary Table S1, and (2) RNA was handled in parallel in the presence and absence of reverse transcriptase. PCR was performed using the following conditions: 95 °C for 5 min (one cycle); 94 °C for 15 s, 58 °C for 30 s, 68 °C for 40–80 s (25–40 cycles); and 75 °C for 5 min (one cycle). Each 20 μL of PCR solution contained 0.5 U of Taq DNA polymerase (TaKaRa Ex TaqHS; Takara Bio, Kusatsu, Japan), 2 μL of 10× PCR buffer containing 20 mmol/L Mg^2+^, 0.2 mmol/L of each deoxyribonucleotide triphosphate, and 0.6 μmol/L of each primer pair. The resulting amplification products were visualized in a 2% agarose gel with 0.5 μg/mL ethidium bromide.

### 2.3. In Situ Hybridization

In situ hybridization experiments were performed as described previously [4,21,22,23,24]. PCR products amplified using specific primer pairs for RAS genes (Supplementary Table S1) were purified and cloned into the pGEM T-easy vector (Promega, Madison, WI, USA) and confirmed by direct sequencing. Digoxigenin-labeled antisense RNA probes were synthesized by in vitro transcription using the digoxigenin-ribonucleic acid (DIG-RNA) Labeling Mix and T7 or SP6 RNA polymerase (Roche, Mannheim, Germany). Frozen blocks of the dissected anterior parts of the tongue embedded in optimum-cutting temperature (OCT) compound (Sakura Fine technical, Tokyo, Japan) were sectioned into 6-μm thick slices, which were mounted on silane-coated glass slides. The cryosections were fixed in 4% paraformaldehyde in phosphate-buffered saline (PBS) for 10 min at room temperature, treated two times with 0.1% diethylpyrocarbonate in PBS for 15 min, washed with 5× saline sodium citrate buffer (SSC) for 15 min at room temperature, and then prehybridized in a hybridization buffer consisting of 50% formamide, 5× SSC, 5× Denhardt’s solution, 500 μg/mL denatured salmon testis DNA and 250 μg/mL denatured baker’s yeast tRNA for 1 h at room temperature. Hybridization was performed for 18 h at 58 °C in a hybridization buffer that included 200 ng/mL antisense (or sense) RNA probe. After hybridization, the sections were washed two times in 5× SSC for 5 min each and two times in 0.2× SSC for 30 min each at 65 °C. Subsequently, the sections were immersed in Tris-buffered saline (TBS) consisting of 50 mmol/L Tris-HCl (pH 7.5), and 150 mmol/L NaCl for 5 min at room temperature, treated with a blocking solution containing 0.5% blocking reagent (Roche) in TBS for 30 min, and applied with anti-digoxigenin Fab fragments-conjugated to alkaline phosphatase (AP; 1:400 dilution; Roche) in blocking solution for 60 min at room temperature. After three washes of 5 min each in Tris-NaCl-Tween 20 (TNT) buffer consisting of 50 mmol/L Tris-HCl (pH 7.5), 150 mmol/L NaCl and 0.05% Tween 20, the sections were immersed in AP buffer comprising 100 mmol/L Tris-HCl (pH 9.5), 100 mmol/L NaCl, and 50 mmol/L MgCl_2_ for 5 min. The signals were developed using 5-bromo-4-chloro-3-indolylphosphate and nitroblue-tetrazolium chloride as chromogenic substrates. Next, the reaction was stopped by washing the slides with Tris-ethylenediaminetetraacetic acid (EDTA) buffer, after which the slides were mounted. The signal specificity of the mRNA for each gene in the taste tissues was tested using a sense RNA probe as a negative control.

### 2.4. Immunohistochemistry

Immunohistochemistry was performed as described previously [4,20]. The dissected tongues of B6, T1R3-GFP or GAD67-GFP mice were fixed in 4% paraformaldehyde in PBS for 45 min at 4 °C. For immunohistochemical analyses for rennin, mice were deprived of water for 47 h (23 h water deprivation, 1 h water drinking, and 23 h water deprivation) before tongue dissection. After dehydration with sucrose solution (10% for 1 h, 20% for 1 h, and 30% for 3 h, at 4 °C), the tongue frozen block was embedded in OCT compound (Sakura Fine technical) and sectioned into 8-μm thick slices, which were mounted on silane-coated glass slides and air-dried. The sections were rinsed with TNT buffer, exposed to 1% blocking reagent (Roche) for 1 h at room temperature, and applied overnight at 4 °C with primary antibodies targeting renin (1:100 dilution; sc-27318; Santa Cruz Biotechnology, Santa Cruz, CA, USA), angiotensinogen (1:100; 11992-1-AP; Protein tech, Chicago, IL, USA), ACE1 (1:100; sc-12187; Santa Cruz Biotechnology), ecto-nucleoside triphosphate diphosphohydrolase-2 (ENTPDase2; 1:100; AF5797; R&D Systems, Minneapolis, MN, USA), epithelial sodium channel α-subunit (αENaC; 1:100; AB3530P; Millipore, Darmstadt, Germany), AT1 (1:100, sc-1173; Santa Cruz Biotechnology) or Gα-gustducin (gustducin; 1:100; sc-395; Santa Cruz Biotechnology) in 1% blocking reagent. After washing with TNT buffer, the tissues were incubated for 2 h at room temperature with peroxidase or alkaline phosphatase-conjugated secondary antibodies (1:500–1000; Jackson ImmunoResearch Laboratories, Philadelphia, PA, USA) in 1% blocking reagent, and this was followed by incubation for 30 min at room temperature with tyramide-Alexa 568 (for GFP-mice) or tyramide-Alexa 488 substrates (TSA kit; Invitrogen) for the detection of renin, ACE1 or angiotensinogen. After washing with TNT, the tissues were incubated with AP buffer for 5 min at room temperature followed by HNPP/FastRed AP substrate (HNPP fluorescent detection kit; Roche) for 40 min at room temperature to detect the signals of the counterpart. The immunofluorescence of labeled cells and GFP fluorescence were observed using a confocal laser scanning microscope (Fluoview FV-1000; Olympus Corp., Tokyo, Japan) and accompanying software. Nomarski images were also obtained in order to visualize individual cells in the taste buds.

To evaluate the number of cells expressing renin, angiotensinogen, ACE1, T1R3-GFP, GAD67-GFP, αENaC, ENTPDase2 and AT1, Nomarski images were overlaid with immunofluorescence (or GFP) images, we then counted the number of positive cells displaying apparent apical processes and/or perinuclear region in each taste bud in horizontal sections of fungiform papillae and circumvallate papillae. Image-Pro Plus v4.0 (Media Cybernetics, Rockville, MD, USA) was used to exclude artifactual signals: cells were considered positive if their signal density was greater than the mean plus two standard deviations (SDs) of that of taste cells in the negative control (primary antibodies omitted). The same cells found on contiguous sections were counted only once.

### 2.5. Quantitative Densitometric Analysis

To examine whether the expression of renin, a principal initiator of the RAS cascade, in taste cells was upregulated in response to dehydration, B6 mice were deprived of water for 47 h (23 h water deprivation, 1 h water drinking, and 23 h water deprivation). The mice had free access to food throughout the procedure. Dissected tongues from water-deprived and non-water-deprived mice (n = 3, each) were fixed at the same time in 4% paraformaldehyde in PBS for 45 min at 4 °C. After dehydration, embedding, and sectioning at 8 μm, both tongue slices were mounted on a single silane-coated glass slide. Then, renin signals were detected by single-molecule immunohistochemistry using CF568-conjugated anti-goat IgG (1:200, Biotium, Hayward, CA, USA) as a secondary antibody instead of a tyramide amplification system, in order to avoid excessive catalyzed reporter deposition of tyramide. All procedures, including image capture, were performed under the same conditions (incubation periods, temperatures, reagent volumes/concentrations, and exposure values for image capture). The taste cell in each section was delineated by comparison with adjacent sections counterstained with gustducin (a type II taste cell marker proposed to be a bitter taste-related G-protein in mouse circumvallate papillae) [25].

Measurement of the total area of a positive cell, and the mean signal intensity was performed using Adobe Photoshop CS6 (Adobe Systems, San Jose, CA, USA) [26]. Regions of interest were drawn around immuno-positive cells in the captured images, and the mean total area and the mean and median pixel intensities of the signals were determined using the Measurement tool in Photoshop. The pixel intensities were normalized against the background by subtracting the value obtained from an area outlying the positive cells in the same section. Two sections of the circumvallate papillae at regular intervals were analyzed for each mouse.

### 2.6. Quantitative PCR (qPCR)

To further examine whether the expression of renin in taste cells was upregulated in response to dehydration, the relative abundance of renin mRNAs in circumvallate taste papillae was examined by using a quantitative PCR method as previously described [24]. The isolated taste buds from each circumvallate papillae of each mouse were pooled (n = 4–7 water-deprived mice, n = 3–7 non-water-deprived mice). As a positive control for renin expression, a 1 mm^3^ block of the kidney was also collected from each mouse (n = 7 and 7, water-deprived and non-water-deprived mice, respectively). Total RNA extraction was performed as described in RT-PCR section. The RNA concentration was measured using NanoDrop ND-1000 (Thermo Fisher Scientific, Waltham, MA, USA). The purity of nucleic acids was assessed by calculating the A260/A280 absorbance ratio. SuperScript VILO Master Mix (cat. no. 11755050, Thermo Fisher Scientific) was used for cDNA synthesis. For quantitative real-time PCR, Fast SYBR Green Master Mix (Applied Biosystems, CA, USA) was used. PCR was performed as follows: 95 °C for 20 s (one cycle); 95 °C for 3 s, 60 °C for 30 s (40 cycles); and 95 °C for 15 s, 60 °C for 1 min, 95 °C for 15 s (one cycle for melting curve analysis) using the ABI StepOnePlus system (Applied Biosystems). Data were analyzed with the StepOne Software (ver. 2.3, Applied Biosystems). The presence of a single amplicon was verified by melting curve analysis, and by agarose gel electrophoresis. Data were obtained from at least three independent experiments, and all reactions were run in triplicate. The quantitative PCR data were normalized using the ΔΔCt method with GAPDH (glyceraldehyde-3-phosphate dehydrogenase) in each sample as reference. ΔΔCt values were calculated by subtracting the average ΔCt of the non-water-deprived samples from each ΔCt of both the non-water-deprived and the water-deprived samples. Fold change from non-water-deprived to water-deprived conditions was calculated as 2^(−ΔΔCT)^. All primer pairs for renin, gustducin, keratin 8, and GAPDH were chosen such that the primers are in separate exons. The PCR primers used for each gene are presented in Supplementary Table S1.

### 2.7. Statistical Analysis

All values are given as the mean ± standard error of the mean. The data were statistically analyzed using Student’s t-test for unpaired samples (Excel; Microsoft Corp., Redmond, WA, USA). *p* < 0.05 was considered statistically significant.

## 3. Results

### 3.1. RT-PCR Reveals Renin-Angiotensin-Related Gene Expression in Mouse Taste Buds

**Figure 1:**
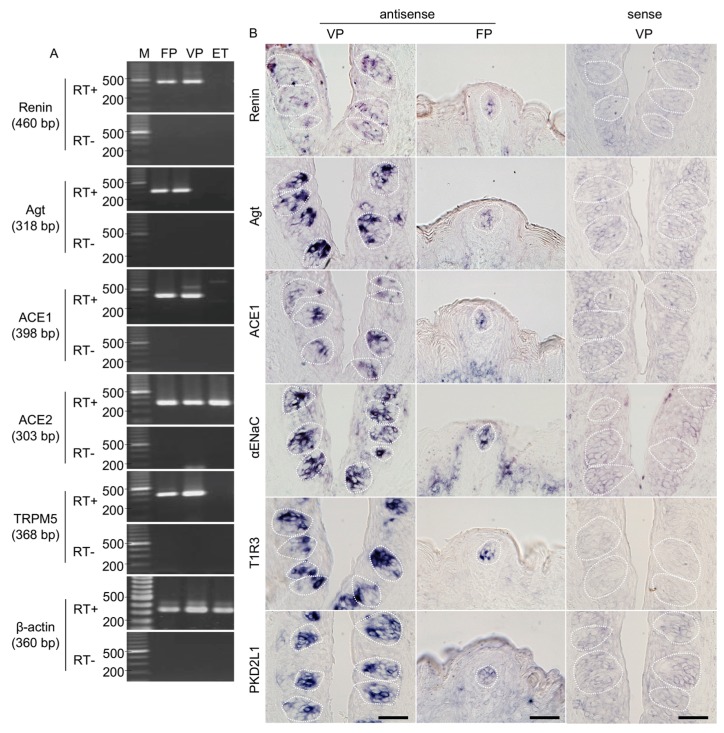
Renin, angiotensinogen (Agt) and angiotensin-converting enzyme-1 (ACE1) mRNAs are expressed in mouse taste bud cells. (***A***) Reverse transcription-polymerase chain reaction (RT-PCR) amplification of renin, Agt, ACE1, ACE2, transient receptor potential channel M5 (TRPM5), and β-actin mRNAs from fungiform papillae (FP), circumvallate papillae (VP), and tongue epithelium devoid of taste cells (ET). RT+ and RT− conditions are, respectively, with and without reverse transcriptase. M (bp): 100 bp marker ladder. (**B**) In situ hybridization detection of renin, Agt, ACE1, epithelial sodium channel α subunit (αENaC), taste receptor type 1 member 3 (T1R3), and polycystic kidney disease 2-like 1 (PKD2L1) in FP and VP of B6 mice. The sense probes served as a negative control. Dotted lines indicate the outlines of taste buds: scale bar, 50 μm.

The expressions of renin, angiotensinogen, ACE1, and ACE2 mRNAs in the taste cells of B6 mice were examined by RT-PCR. As shown in Figure 1A, bands of the correct size (460 bp for renin, 318 bp for angiotensinogen, 398 bp for ACE1, and 303 bp for ACE2) were evident in taste papillae. Renin, angiotensinogen, and ACE1 mRNAs were expressed in fungiform and circumvallate papillae but not in tongue epithelium devoid of taste buds. Similarly, RT-PCR products for a taste cell marker, transient receptor potential channel M5 (TRPM5; 368 bp) [27,28,29,30], were also found in fungiform and circumvallate papillae but not in tongue epithelium devoid of taste buds. ACE2 mRNA was expressed in all tissues. As a positive control, β-actin mRNA (360 bp) was also detected in all tissues. All control experiments in which the reverse transcriptase enzyme was omitted (RT-) yielded negative results.

### 3.2. Renin-Angiotensin-Related Genes Are Localized to a Subset of Taste Bud Cells

In situ hybridization experiments detected renin, angiotensinogen, and ACE1 mRNA in a subset of cells in the fungiform and circumvallate papillae of mice but not in surrounding epithelial cells (Figure 1B). Comparable results were obtained for three markers of taste cells: αENaC (an amiloride-sensitive salt taste receptor subunit candidate) [31], T1R3 (a sweet/umami taste receptor component) [32,33] and polycystic kidney disease 2-like 1 (PKD2L1; a sour taste-related molecule) [34,35] (Figure 1B). Control hybridizations using sense probes for renin, angiotensinogen, ACE1, αENaC, T1R3, and PKD2L1 were negative. These results, together with the RT-PCR data, strongly suggest that renin, angiotensinogen, and ACE1 are expressed in mouse taste bud cells of both the anterior and posterior tongue.

### 3.3. Renin-Angiotensin-Related Proteins Are Co-Expressed with T1R3 or αENaC in Taste Bud Cells

**Figure 2:**
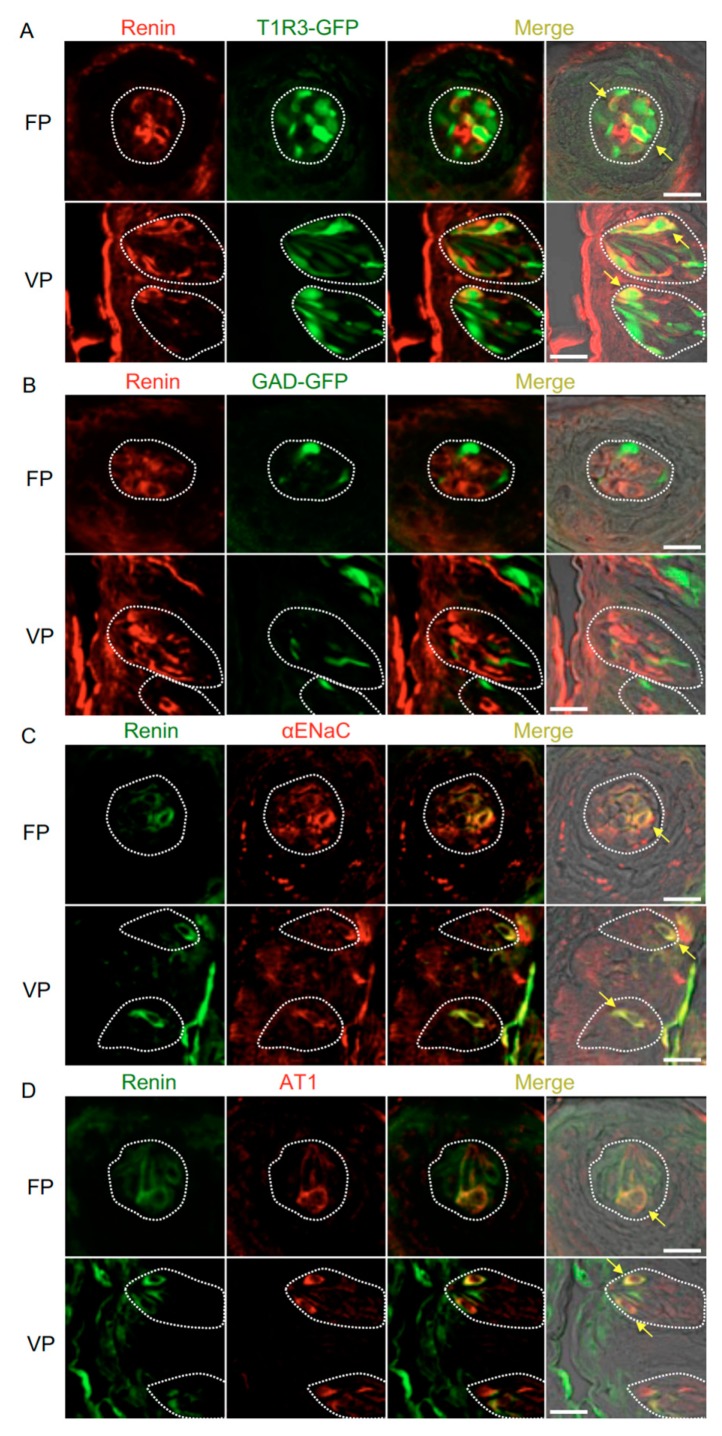
Co-expression of renin with taste receptor family 1 member 3 (T1R3), glutamate decarboxylase 67 (GAD), epithelial sodium channel α subunit (αENaC) or angiotensin II type 1 receptor (AT1) in taste bud cells. (**A**) Expression of renin in fungiform papillae (FP) and circumvallate papillae (VP) of T1R3-green fluorescent protein (GFP) mice. (**B**) Expression of renin in FP and VP of GAD67-GFP mice. (**C**) Co-expression of renin with αENaC in FP and VP of B6 mice. (**D**) Co-expression of renin with AT1 in FP and VP of B6 mice. Immunostaining for renin is shown in red (**A**,**B**) or green (**C**,**D**). GFP fluorescence in T1R3-GFP and GAD67-GFP mice is shown in green (**A**,**B**). Immunostaining for αENaC and AT1 is shown in red (**C**,**D**). The last merge panels: Nomarski images were overlaid with immunofluorescence (or GFP) merge images. Arrows indicate renin-expressing taste cells that co-express T1R3, αENaC, or AT1. Dotted lines indicate the outlines of taste buds: scale bar, 25 μm.

Immunohistochemistry experiments detected renin, ACE1, and angiotensinogen in some spindle-shaped taste cells of the fungiform and circumvallate papillae of the mouse but not in surrounding tissues or gustatory nerves. Notably, co-expression of renin and taste cell markers was observed (Figure 2 and Table 1). In both the fungiform and circumvallate papillae, a subset of renin-positive cells expressed αENaC (ENaC/renin: 83.9% in fungiform papillae and 88.1% in circumvallate papillae) and T1R3 (as marked by T1R3-GFP; T1R3/renin: 54.7% in fungiform papillae and 49.2% in circumvallate papillae). Renin-expressing cells also showed immunoreactivity for AT1 (AT1/renin: 72.0% in fungiform papillae and 80.0% in circumvallate papillae) [4], but renin expression was not found in sour/type III cells as marked by GAD67-GFP (GAD/renin: 0% in both fungiform and circumvallate papillae).

**Figure 3:**
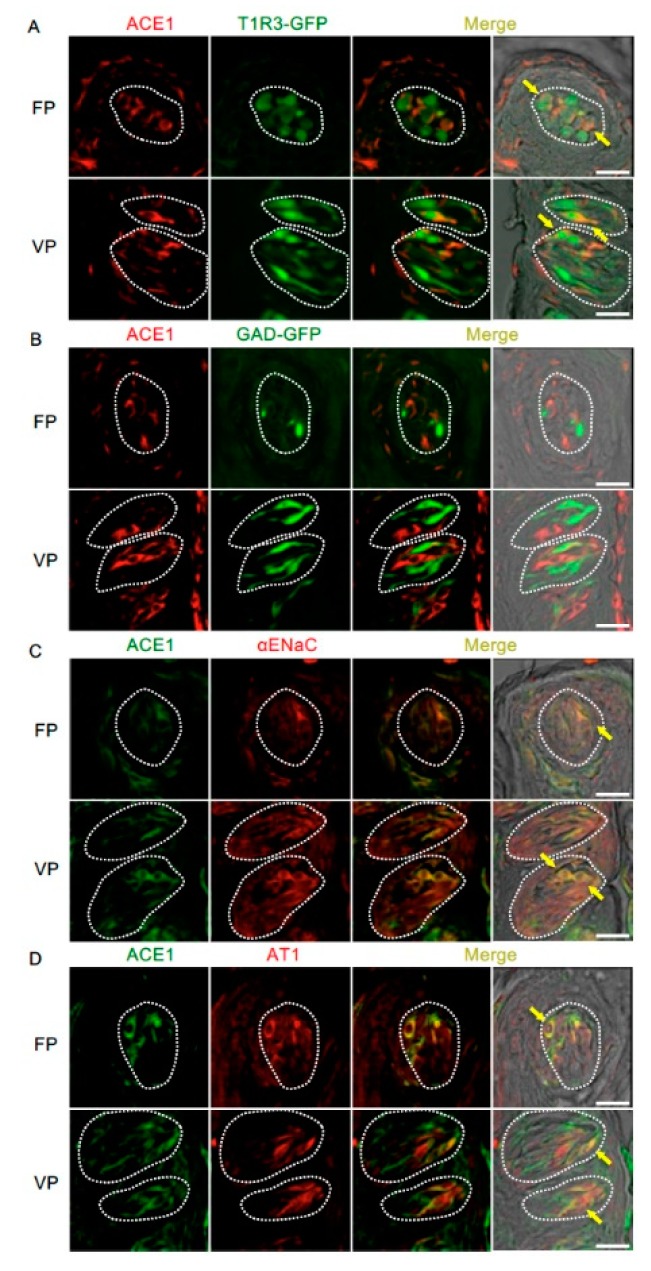
Co-expression of angiotensin-converting enzyme-1 (ACE1) with taste receptor type 1 member 3 (T1R3), glutamate decarboxylase 67 (GAD), epithelial sodium channel α subunit (αENaC) or angiotensin II type 1 receptor (AT1) in taste bud cells. (**A**) Expression of ACE1 in fungiform papillae (FP) and circumvallate papillae (VP) of T1R3-green fluorescent protein (GFP) mice. (**B**) Expression of ACE1 in FP and VP of GAD67-GFP mice. (**C**) Co-expression of ACE1 with αENaC in FP and VP of B6 mice. (**D**) Co-expression of ACE1 with AT1 in FP and VP of B6 mice. Immunostaining for ACE1 is shown in red (**A**,**B**) or green (**C**,**D**). GFP fluorescence in T1R3-GFP and GAD67-GFP mice is shown in green (**A**,**B**). Immunostaining for αENaC and AT1 is shown in red (**C**,**D**). The last merge panels: Nomarski images were overlaid with immunofluorescence (or GFP) merge images. Arrows indicate ACE1-expressing taste cells that co-express T1R3, αENaC, or AT1. Dotted lines indicate the outlines of taste buds: scale bar, 25 μm.

The co-expression of ACE1 and taste cell markers was also examined (Figure 3 and Table 2). ACE1-positive cells expressed αENaC (ENaC/ACE1: 71.9% in fungiform papillae and 91.3% in circumvallate papillae) and T1R3 (T1R3/ACE1: 70.0% in fungiform papillae and 57.5% in circumvallate papillae). ACE1-expressing cells also exhibited positivity for AT1 (AT1/ACE1: 81.3% in fungiform papillae and 79.7% in circumvallate papillae). ACE1 expression was rarely observed in GAD67-expressing cells (GAD/ACE1: 0% in fungiform papillae and 2.7% in circumvallate papillae).

**Figure 4:**
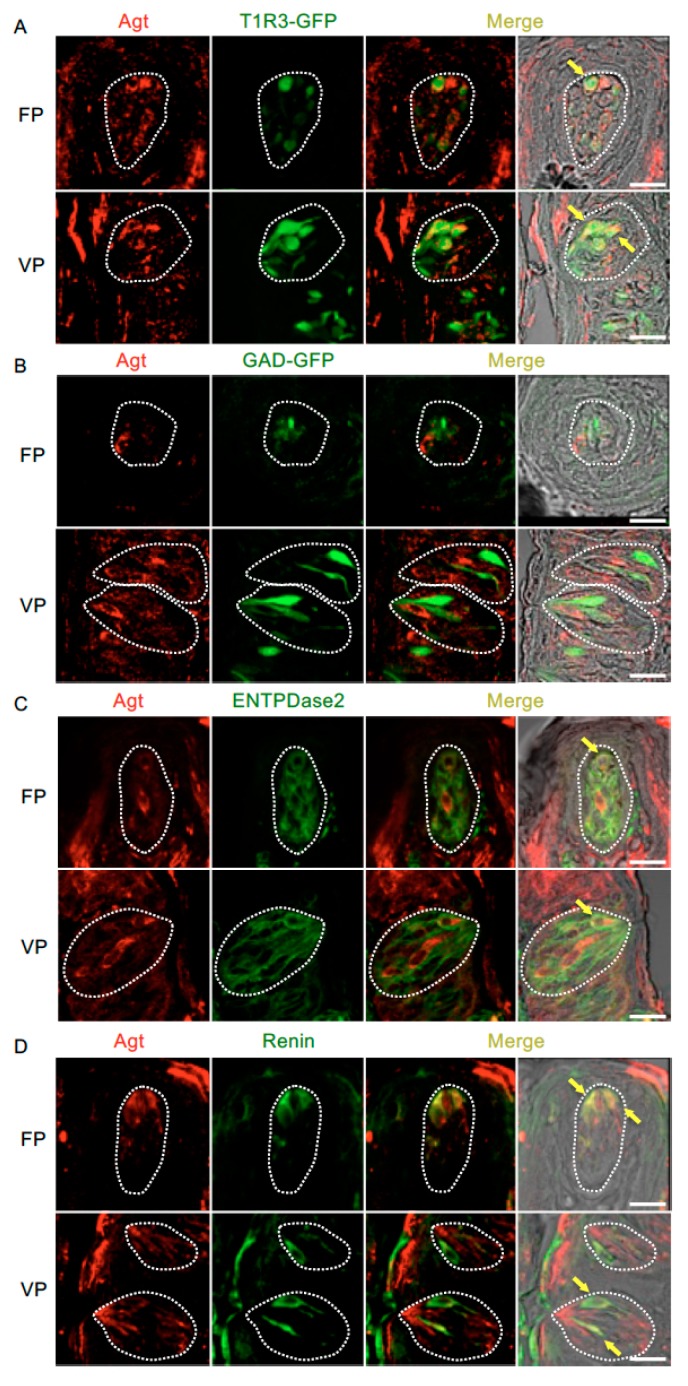
Co-expression of angiotensinogen (Agt) with taste receptor type 1 member 3 (T1R3), glutamate decarboxylase 67 (GAD), nucleoside triphosphate diphosphohydrolase-2 (ENTPDase2) and renin in taste bud cells. (**A**) Expression of Agt in fungiform papillae (FP) and circumvallate papillae (VP) of T1R3-green fluorescent protein (GFP) mice. (**B**) Expression of Agt in FP and VP of GAD67-GFP mice. (**C**) Co-expression of Agt with ENTPDase2 in FP and VP of B6 mice. (**D**) Co-expression of Agt with renin in FP and VP of B6 mice. Immunostaining for Agt is shown in red. GFP fluorescence in T1R3-GFP and GAD67-GFP mice and immunostaining for ENTPDase2 and renin are shown in green. The last merge panels: Nomarski images were overlaid with immunofluorescence (or GFP) merge images. Arrows indicate Agt-expressing taste cells that co-express T1R3, ENTPDase2, or renin. Dotted lines indicate the outlines of taste buds: scale bar, 25 μm.

Figure 4 and Table 3 present data regarding the co-expression of angiotensinogen and taste cell markers. The majority of angiotensinogen-positive cells expressed T1R3 (T1R3/angiotensinogen: 73.0% in fungiform papillae and 70.7% in circumvallate papillae) and renin (renin/angiotensinogen: 75.8% in fungiform papillae and 70.6% in circumvallate papillae). A subset of cells expressing ENTPDase2, a type I taste cell marker [36], showed positive signals for angiotensinogen (ENTPDase2/angiotensinogen: 37.5% in fungiform papillae and 34.1% in circumvallate papillae). Angiotensinogen was not observed in GAD67-expressing cells (GAD/angiotensinogen: 0% in both fungiform and circumvallate papillae).

**Figure 5:**
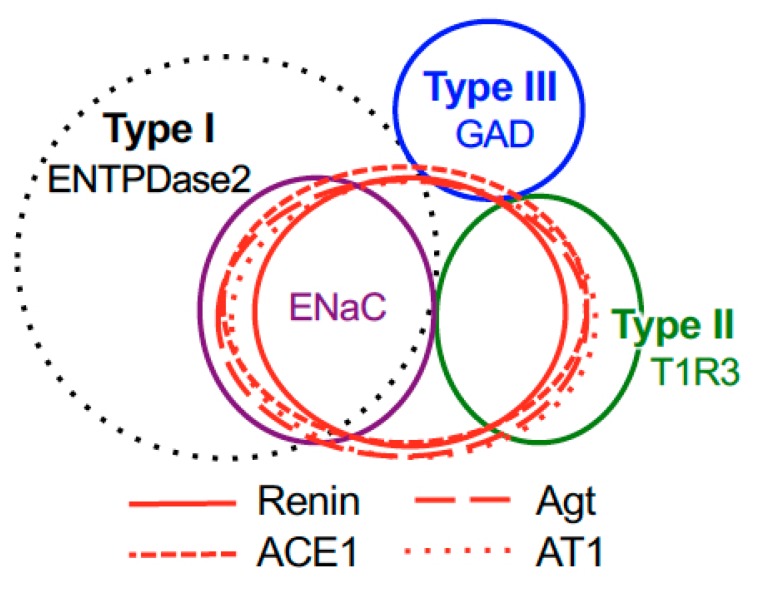
Summary of the patterns of co-expression between renin, angiotensinogen (Agt), angiotensin-converting enzyme-1 (ACE1), angiotensin II type 1 receptor (AT1) and type-specific markers of taste cells [nucleoside triphosphate diphosphohydrolase-2 (ENTPDase2) for cell type I, taste receptor type 1 member 3 (T1R3) for cell type II, and glutamate decarboxylase 67 (GAD) for cell type III] in both the fungiform and circumvallate papillae of mice.

The inverse co-expression ratios (renin, ACE1, and angiotensinogen/taste cell markers) are shown in Table 1, Table 2 and Table 3. A summary of the expression patterns of renin, ACE1, angiotensinogen, and taste cell markers in the fungiform and circumvallate papillae is shown in Figure 5.

### 3.4. Renin Expression Is Upregulated in the Taste Buds Cells of Water-Deprived Mice

**Figure 6:**
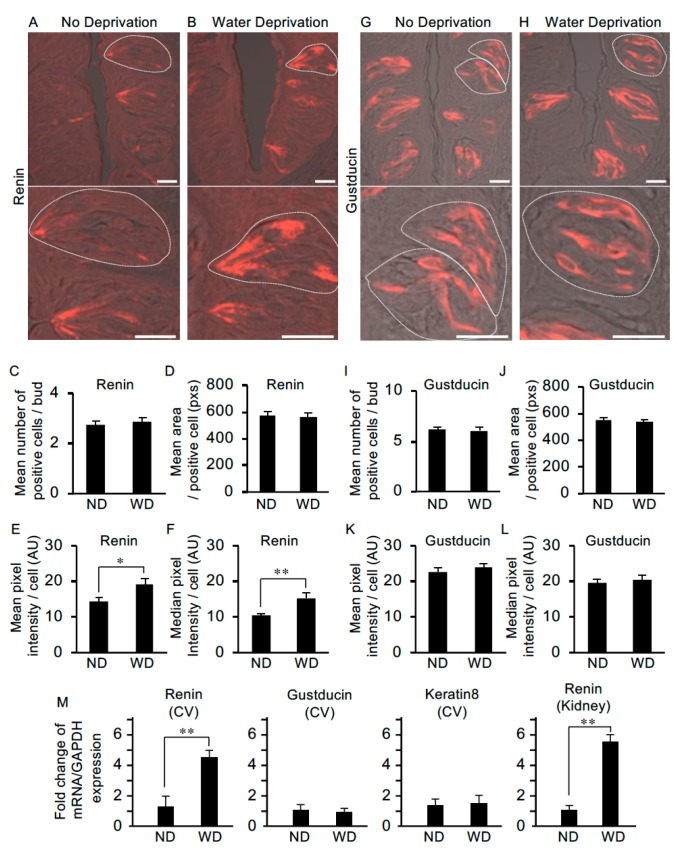
The level of renin expression in taste cells is upregulated after water deprivation. (**A**) In non-deprived (control) mice, renin was expressed at the apical process regions of taste cells and more weakly in the cell body regions (lower image: higher magnification of the upper image). (**B**) In mice deprived of water for 47 h, renin expression was observed throughout some cells, i.e., from apical to basal regions. Water deprivation did not change the mean number of renin-positive cells per taste bud [n = 28 taste buds from three non-water deprived mice (ND), n = 27 taste buds from three water-deprived mice (WD)] (**C**) or the mean area of renin-positive cells (n = 28 taste cells from ND, n = 28 taste cells from WD) (pixels: pxs) (**D**). However, significant differences were detected in the mean pixel intensity (**E**) and median pixel intensity (**F**) of renin-positive cells (arbitrary unit: AU). Gustducin immunoreactivity was detected throughout positive cells from the apical to the basal regions in both non-water-deprived mice (**G**) and water-deprived mice (**H**). Water deprivation did not change the mean number of gustducin-positive cells per taste bud (n = 23 taste buds from ND, n = 23 taste buds from WD) (**I**), mean area of gustducin-positive cells (n = 32 taste cells from ND, n = 38 taste cells from WD) (pixels: pxs) (**J**), mean pixel intensity of gustducin-positive cells (**K**) or median pixel intensity (**L**) of gustducin-positive cells (arbitrary unit: AU). (**M**) Quantification of mRNA expression of renin, gustducin and keratin 8 by real-time PCR in the circumvallate taste papilla (CV) and Kidney (as the control tissue for renin expression) from ND and WD. Data were obtained from at least three independent experiments (n = 3–7 mice) per group each PCR assays were performed in triplicate. The quantitative PCR results were normalized using the ΔΔCt method with GAPDH (glyceraldehyde-3-phosphate dehydrogenase) in each sample as the reference, and shown as fold change of mRNA expression compared to ND. * p < 0.05, ** p < 0.01 for comparisons between ND and WD groups (Student’s t-test). All data are presented as the mean ± standard error of the mean. Dotted lines indicate the outlines of taste buds: scale bar, 25 μm.

To examine whether water deprivation leads to changes in the abundance of renin, we performed immunohistochemistry and quantitative image analyses using circumvallate papillae from non-deprived and water-deprived mice. In non-deprived mice (used as the control), immunoreactivity for renin was observed in the apical process regions of taste cells with weaker expression in the cell body regions (Figure 6A). In water-deprived mice, immunoreactivity for renin was observed throughout some cells, i.e., from apical to basal regions (Figure 6B). There were no significant differences between non-deprived and water-deprived mice in the mean number of positive cells per bud [2.75 ± 0.2 (n = 28) vs. 2.85 ± 0.2 (n = 27)] or the mean area (pixels) of a positive cell [576.7 ± 25.0 (n = 28) vs. 563.2 ± 27.4 (n = 28)] (Figure 6C,D). However, significant differences were observed between control and water-deprived mice in both the mean pixel intensity (arbitrary units) [14.5 ± 0.9 (n = 28) vs. 19.2 ± 1.5 (n = 28), p < 0.05] and the median pixel intensity (arbitrary units) [10.3 ± 0.6 (n = 28) vs. 15.4 ± 1.4 (n = 28), p < 0.01] of the immuno-positive cells (Figure 6E,F).

We also examined differences in the abundance of gustducin protein (a taste-specific G-protein mostly expressed in bitter taste cells in mouse circumvallate papillae) in taste cells between control and water-deprived mice. Immunoreactivity for gustducin was observed throughout the apical-to-basal regions of a subset of taste cells in both the non-deprived and water-deprived groups (Figure 6G,H). We observed no significant differences between the non-deprived and water-deprived groups in the mean number of positive cells per taste bud [6.13 ± 0.3 (n = 23) vs. 6.09 ± 0.3 (n = 23)], the mean area of a positive cell [548.5 ± 20.4 (n = 32) vs. 532.8 ± 20.5 (n = 38)], the mean pixel intensity of a positive cell [22.8 ± 1.1 (n = 32) vs. 23.8 ± 1.2 (n = 38)] or the median pixel intensity of a positive cell [19.6 ± 1.1 (n = 32) vs. 20.6 ± 1.2 (n = 38)] (Figure 6I–L).

Next, we asked whether renin mRNA expression in circumvallate taste bud cells is upregulated after water-deprivation by using quantitative PCR. mRNA levels of genes were normalized for GAPDH as the endogenous reference gene and shown as fold change of mRNA expression compared to non-water-deprived groups (Figure 6M). Statistical analysis by *t*-test revealed a significant difference in relative renin mRNA expression in circumvallate taste buds between control and water-deprived mice [1.36 ± 0.62 (n = 3 mice) vs. 4.60 ± 0.46 (n = 4), p < 0.01]. In the Kidney as the control tissue for renin expression, renin mRNA was significantly increased in the water-deprived group compared with the non-water-deprived group [1.11 ± 0.22 (n = 7) vs. 5.53 ± 0.51 (n = 7), p < 0.01) (Figure 6M). We also performed quantitative PCR analysis of gustducin and keratin 8 (a pan-taste cell marker) in the circumvallate taste bud cells, and observed no significant differences in the mRNA expression between control and water-deprived groups [gustducin: 1.14 ± 0.33 (n = 5) vs. 1.02 ± 0.20 (n = 7), keratin 8: 1.37 ± 0.43 (n = 6) vs. 1.51 ± 0.52 (n = 6)] (Figure 6M). Together, these results suggest that renin expression is upregulated in taste cells in response to dehydration.

## 4. Discussion

In the present study, we found that three RAS components, namely renin, ACE1, and angiotensinogen, were present in the taste buds of fungiform and circumvallate papillae and co-expressed with αENaC (a salt taste receptor), T1R3 (a sweet taste receptor component) [31,32,33] and AT1 [4]. Furthermore, significantly increased levels of renin expression were observed in taste cells after water deprivation of the mouse. These results suggest that the taste organ contains a local RAS that may be capable of producing AngII within the taste buds. AngII may function as a modulator of amiloride-sensitive salt and sweet taste sensitivities via AT1 in an autocrine and paracrine manner.

In animals with AngII-dependent hypertension, the intrarenal AngII level is higher than that which can be explained on the basis of equilibration with circulating AngII [12]. This suggests that AngII can be synthesized within the kidney. Indeed, all components of the RAS are present in the distal nephron [11,37]. Principal cells in the connecting tubule and the cortical collecting duct abundantly express renin, a key initiator of the RAS, which cleaves the N-terminal end of angiotensinogen to generate AngI in the tubular fluid. Renin immunolocalizes predominately to the apical side of the cytoplasm of principal cells. Quantitative histological analysis revealed that mice exhibited minimal renin immuno-staining (below the detection level) in the connecting tubule under high sodium diets, in contrast, overnight sodium restriction led to a marked increase in the number of the renin-positive connecting tubule cells. Furthermore, the amount of renin mRNA was shown to increase in response to sodium restriction [11,37]. In our study, renin immunostaining predominated at the apical regions of taste cells, which face the oral cavity and are available to sense various chemical compounds in saliva or foods (Figure 6A). Moreover, significantly increased levels of renin in taste cells were observed in water-deprived mice (Figure 6E,F,M), which may be consistent with the results observed in connecting tubule cells in the kidney [11]. These results indicate that renin synthesis initiating the RAS cascade occurs in taste cells and may be regulated in response to factors in the oral environment such as sodium concentration (osmolality) in the saliva or chemical compounds in foods, which are sensed at the apical regions of the taste cells.

A previous study showed that amiloride-sensitive NaCl taste responses were suppressed 10–30 min after intraperitoneal injection of AngII, suggesting that systemic AngII produced by the circulating RAS is able to modulate NaCl taste responses in taste cells [4]. Thus, there may be two pathways regulating taste, namely the circulating RAS and the local RAS. What would be the advantage of the co-existence of these two RASs? One possibility is that the local RAS assists in maintaining the constancy of body NaCl balance through acute changes in AngII production in response to fasting or random perturbations in the oral cavity during feeding and drinking. In other words, the local RAS could help to avert decreases in body sodium levels or prevent excessive sodium/calorie consumption independently of changes in body NaCl balance. However, once NaCl balance has been changed, the distal nephron NaCl concentration would serve to regulate the circulating RAS, which supports the maintenance of prolonged deviations of body NaCl concentration from the normal set point [5]. Such temporally integrated regulation of taste sensitivity by local AngII (short-term feedforward regulation predicting changes in body fluid composition) and systemic AngII (long-term negative feedback regulation in response to changes in body salt/water balance) may play an important role in sodium/energy homeostasis. ACE2, which degrades Ang II to Ang (1–7) to oppose the actions of Ang II, is present not only in fungiform and circumvallate papillae but also in the tongue epithelium (Figure 1A). This observation suggests that AngII generated locally in taste buds can be rapidly degraded by ACE2, which would support the hypothesis of short-term regulation of taste sensitivity by a local RAS.

Multiple lines of evidence from studies using molecular approaches indicate that the basic taste qualities (sweet, salty, bitter, sour, and umami) are mediated by distinct taste cells expressing unique taste receptors [38]. Sweet, umami and bitter substances activate G-protein coupled receptors (T1R2+T1R3 for sweet [32,33], T1R1+T1R3 for umami [33] and T2Rs for bitter [39]) and subsequent common signaling pathways involving phospholipase C-β [28], the type 3 inositol 1,4,5-trisphosphate receptor [40] and TRPM5 [27,28,29,30]. Salty and sour substances are believed to activate channel-type receptors (ENaC for amiloride-sensitive salt taste [31,41] and PKD2L1/1L3 for sour taste [34,35]). Each of these taste receptors is expressed in a different set of taste cells [38]. The present study demonstrated that all three of the RAS components studied are co-expressed with αENaC or T1R3 but not GAD67, suggesting that taste-regulated AngII production by the local RAS may be mediated by αENaC-expressing, amiloride-sensitive salt taste cells, and T1R3-expressing sweet taste cells. The expression patterns of the RAS components in the taste organ may relate to previous observations that AngII suppresses amiloride-sensitive salt taste responses and enhances sweet taste responses without any effects on bitter, umami and sour responses [4]. The relationship between salt and sweet preferences via AngII signaling, analogous to that between reduced salt-sensitive neural responses and increased sugar-sensitive neural responses to dietary NaCl in the rat nucleus of the solitary tract after intracerebroventricular renin infusion on chronic deoxycorticosterone acetate (DOCA, the precursor of aldosterone) treatment [42], or that between sodium and glucose absorption via sodium-glucose cotransporter-1 in the small intestine [43], may optimize sodium and calorie intake via the taste system.

The macula densa is a group of 15–20 epithelial cells in the distal convoluted tubule of the kidney. These cells play a critical role in sensing changes in tubular fluid composition and sending signals to the juxtaglomerular apparatus that controls renin release [44]. It has been shown that increasing the NaCl concentration at the macula densa suppresses renin release, whereas reducing the NaCl concentration results in a prompt stimulation of renin release [45]. NaCl sensing by the macula densa involves apical NaCl transport mechanisms, including the furosemide-sensitive Na^+^/K^+^/2Cl^−^cotransporter (NKCC), which is the primary NaCl entry mechanism [46,47]. The apical membranes of macula densa cells also express Na^+^/H^+^ exchanger (NHE), which participates in Na^+^ transport and the regulation of intracellular pH and cell volume [48,49]. It has been reported that NHE1 and NHE3 were detected in taste receptor cells [50]. It is possible that NKCC or NHE may participate in sensing changes in NaCl concentration at the cell membrane of taste cells.

It is generally accepted that an elevation in the circulating level of AngII inhibits renin secretion from the juxtaglomerular apparatus of the kidney. However, intrarenal AngII increases renin mRNA and protein levels in the distal nephron [51], indicating positive-feedback regulation of intrarenal RAS by AngII. In experiments involving renal cross-transplantation between global AT1-knockout mice and wild-type controls, AngII was shown to cause hypertension through stimulation of AT1 receptors in the kidney [52]. Overexpression of renin in the collecting duct caused spontaneous hypertension [53]. The Aldosterone/NaCl-induced RAS functional impairment also, not only caused a reduction of the salt taste sensitivity, but also salt-sensitive hypertension in rat [54]. These results suggest that intrarenal RAS contributes to the pathogenesis of hypertension. The existence of such a positive-feedback mechanism in the taste organ potentially would have broad implications, since continuous activation of the taste organ RAS might be associated with hypertension induced by excessive salt consumption through sustained low salt taste sensitivity.

## 5. Conclusions

We have demonstrated that the taste organ has three major components of the RAS, namely renin, angiotensinogen, and ACE1, which would enable AngII to be produced locally in the taste buds. Expression analyses showed that the RAS components are co-expressed with αENaC or T1R3 in a subset of taste cells. Renin immunoreactivity was detected at the apical regions of taste cells, which face the oral cavity and thus are exposed to various chemical compounds in saliva or food. Furthermore, renin synthesis in taste tissue was significantly upregulated in response to water deprivation. Taken together, these results suggest the existence of a previously unidentified local RAS in the taste organ. The specific reduction of amiloride-sensitive salt taste sensitivity and enhancement of sweet taste sensitivity may be mediated by both locally-produced AngII (temporal feedforward regulation) and circulating AngII (continuous negative feedback regulation). Such an integrated regulation of peripheral taste sensitivity by AngII may play an important role in sodium/calorie homeostasis.

## Figures and Tables

**Table 1:** Co-expression ratio of Renin and taste cell markers in fungiform (FP) and circumvallate papillae (VP) in mice.

	FP		VP			FP		VP	
T1R3/Renin	54.7%	(29/53, n = 12)	49.2%	(31/63, n = 17)	Renin/T1R3	39.7%	(29/73, n = 12)	36.0%	(31/86, n = 17)
GAD/Renin	0%	(0/33, n = 8)	0%	(0/42, n = 11)	Renin/GAD	0%	(0/13, n = 8)	0%	(0/33, n = 11)
ENaC/Renin	83.9%	(26/31, n = 10)	88.1%	(37/42, n = 11)	Renin/ENaC	92.9%	(26/28, n = 10)	86.0%	(37/43, n = 11)
AT1/Renin	72.0%	(18/25, n = 9)	80.0%	(32/40, n = 11)	Renin/AT1	72.0%	(18/25, n = 9)	91.4%	(32/35, n = 11)

AT1: angiotensin II type 1 receptor; ENaC: epithelial sodium channel α-subunit; GAD: glutamate decarboxylase; T1R3: taste receptor type 1 member 3. The number of protein1 + protein2 doubly labeled cell/number of protein2 positive cell and n = number of taste buds examined are shown in parentheses.

**Table 2:** Co-expression ratio of ACE1 and taste cell markers in fungiform (FP) and circumvallate papillae (VP) in mice.

	FP		VP			FP		VP	
T1R3/ACE	70.0%	(35/50, n = 11)	57.5%	(42/73, n = 20)	ACE/T1R3	58.3%	(35/60, n = 11)	46.2%	(42/91, n = 20)
GAD/ACE	0%	(0/38, n = 11)	2.7%	(2/75, n = 16)	ACE/GAD	0%	(0/15, n = 11)	4.3%	(2/47, n = 16)
ENaC/ACE	71.9%	(23/32, n = 7)	91.3%	(42/46, n = 10)	ACE/ENaC	85.2%	(23/27, n = 7)	93.3%	(42/45, n = 10)
AT1/ACE	81.3%	(26/32, n = 7)	79.7%	(63/79, n = 13)	ACE/AT1	96.3%	(26/27, n = 7)	98.4%	(63/64, n = 13)

ACE1: angiotensin-converting enzyme-1; AT1: angiotensin II type 1 receptor; ENaC: epithelial sodium channel α-subunit; GAD: glutamate decarboxylase; T1R3: taste receptor type 1 member 3. The number of protein1 + protein2 doubly labeled cell/number of protein2 positive cell, and n = number of taste buds examined are shown in parentheses.

**Table 3:** Co-expression ratio of Agt and taste cell markers in fungiform (FP) and circumvallate papillae (VP) in mice.

	FP		VP			FP		VP	
T1R3/Agt	73.0%	(27/37, n = 7)	70.7%	(41/58, n = 14)	Agt/T1R3	60.0%	(27/45, n = 7)	37.6%	(41/109, n = 14)
GAD/Agt	0%	(0/10, n = 4)	0%	(0/56, n = 17)	Agt/GAD	0%	(0/8, n = 4)	0%	(0/68, n = 17)
ENTPD2/Agt	37.5%	(9/24, n = 9)	34.1%	(15/44, n = 14)	Agt/ENTPD2	8.7%	(9/103, n = 9)	7.9%	(15/189, n = 14)
Renin/Agt	75.8%	(25/33, n = 9)	70.6%	(24/34, n = 9)	Agt/Renin	92.6%	(25/27, n = 9)	85.7%	(24/28, n = 9)

Agt: angiotensinogen; ENTPDase2: ecto-nucleoside triphosphate diphosphohydrolase-2; GAD: glutamate decarboxylase; T1R3: taste receptor type 1 member 3. The number of protein1 + protein2 doubly labeled cell/number of protein2 positive cell, and n = number of taste buds examined are shown in parentheses.

## Supplementary Materials

The following are available online at https://www.mdpi.com/2072-6643/11/9/2251/s1, Table S1: PCR primers.
