# Supplementary material for: Expression of Renin-Angiotensin System Components in the Taste Organ of Mice
Source: Nutrients. 2019 Sep 19;11(9):2251. doi: 10.3390/nu11092251 (PMC6770651; doi:10.3390/nu11092251)
Supplement: Supplementary file 1 [file nutrients-11-02251-s001.pdf]

Supplemental table. Nucleotide sequences for the primers used in RT-PCR, *in situ* hybridization and qPCR experiments.

Gene	Accession No.	Analysis	Forward	Reverse	Region
Renin	NM_031192	RT-PCR	aagaaggctgtgcggtagtg	tccctatgttggggacaaag	868–1327
		ISH	accttcagtctcccaacacg	tgtgcacagcttgtctctcc	87–1109
		qPCR	ccgacatttcctttgacctg	tgtgcacagcttgtctctcc	1015-1109
Agt	NM_007428	RT-PCR	ccgaggatcctacaatctgc	ctaggcacaaggcctcacac	1193–1510
		ISH	ccccttccatctcctttacc	cctctcctgctttgagttcg	152–1344
ACE1	NM_207624	RT-PCR	accgagaacaggagacatgg	aggaggaaggctcagctagg	3678–4075
		ISH	atggaagcatcaccaaggag	cttccttcccctctgtttcc	3283–4454
ACE2	NM_001130513	RT-PCR	tgatgtctttggcctgaatg	ggaagatgacaagtgctttgc	2466–2768
TRPM5	NM_020277	RT-PCR	caggaggcacctactcaagc	atccagctgtccagtccaag	3458–3825
β-actin	NM_007393	RT-PCR	ggttccgatgccctgaggctc	acttgcggtgcacgatggagg	840–1199
αENaC	NM_011324	ISH	ctaatgatgctggaccacacc	aaagcgtctgttccgtgatgc	539–1094
T1R3	NM_031872	ISH	tgctgctatgactgcgtggac	aagaagcacatagcacttggg	1583–2488
PKD2L1	NM_181422	ISH	gaaagagcgggtttctgatg	ccctcagttccagctagtcg	1962–3159
Gustducin	NM_001081143	qPCR	agggcatctgaataccagctcaa	ctgatctctggccacctacatcaa	533-728
Keratin 8	NM_031170	qPCR	tgaacaacaagttcgcctcctt	gctcctcgacgtcttctgct	414-523
GAPDH	NM_001289726	qPCR	tgtgtccgtcgtggatctga	ttgctgttgaagtcgcaggag	803-952

ISH: *in situ* hybridization; RT-PCR: reverse transcription-polymerase chain reaction; qPCR: quantitative polymerase chain reaction.
